# The Treatment of Insanity: Existing Defects and their Remedy

**Published:** 1907-04-20

**Authors:** 


### THE TREATMENT OF INSANITY.

### Existing Defects and their Remedy.

### II. THE PUBLIC ASYLUM, THE REGISTERED HOSPITAL, AND THE LICENSED HOUSE.

So long as mental disorder is not very pronounced, the neurologist consultant, who is usually first called upon to assist the general practitioner, is willing to remain in charge of the case; but if the insanity is unmistakable, he commonly retires from the case, and the alienist consultant comes upon the scene. The alienist usually has to consider, and determine on, the propriety, first, of allowing the patient to remain at home or of removing him; and further, if the second alternative is chosen, of determining the destination of the patient. He does not entertain the project of a sea voyage, nor does he recommend a nursing home, except for some purely temporary reason and as a transient measure. The alternatives that occur to him are travelling or residing with a selected companion; private or family care; or entrance into an institution. In selected cases, which are very few, however, the first course is judicious and efficient. In other selected cases the second course is, as we have already seen, attended by excellent results. Whichever course is selected, the choice of the companion or host is of the greatest importance. The residue, which comprises by far the greatest number of cases of pronounced mental disorder, must be sent to an "institution for the insane" specially adapted, equipped, and staffed for the purpose of receiving persons of unsound mind.

Of these institutions there are in this country three kinds—the public asylum, the registered hospital, and the licensed house. The first is mainly for the "pauper" class," a term which has in this connection its strictly legal meaning, and merely signifies a person who is unable to support himself, or whose relatives are unable to support him. It does not at all necessarily connote the undesirable qualities that are apt to be associated with the term "pauper." Many most respectable persons—artisans, clerks, governesses, and professional men—and their relatives, become "paupers" of necessity when they become insane. Many of the public asylums have now a private wing in which patients are received for sums which wholly cover the cost of their maintenance—a very excellent provision—and some public asylums have a private wing in which they receive patients for sums much in excess of the cost of maintenance, so making a large profit—a provision the excellence of which, as a form of municipal trading, is open to considerable doubt.

The second class of institution consists of the registered hospital, founded, built, and usually to some extent endowed, by private persons for the reception, at unremunerative rates, of persons who have been gently nurtured and are unable to afford the fees of licensed houses. These institutions are managed by committees who give their time and services gratuitously, and the officers who perform the medical and administrative work are paid fixed, and usually very liberal, salaries. In this way it was proposed to eliminate the "principle of profit" which, Lord Shaftesbury declared, "vitiates the whole thing." Whether the noble-minded peer who procured the magna charter of the insane, the Lunacy Acts, 1845, was right or wrong, we need not inquire, for in spite of his legislation and of the well-meant efforts of many other philanthropists, the "principle of profit" has not only not been removed, but has extended from licensed houses to registered hospitals, and from registered hospitals to public asylums. It is hard to eradicate one of the fundamental instincts of humanity; and indeed, "the principle of profit" is a necessary part of their being. They are most of them endowed, but the endowments are not nearly enough to pay the current expenses. For these it is necessary to depend mainly upon the payments made by the patients; and manifestly, the larger these payments are in the aggregate, the greater is the success of the administration. The men at the head of these institutions share with the rest of Western humanity the desire to be successful, and to be known to be successful. If the institution was unable to pay its way it would have to be closed, and the staff must be dispensed with. The better it pays, the more money there is to spend on improvements—and improvements are always needed. The accommodation soon becomes insufficient, and the buildings must be extended. The larger number of patients necessitates a new kitchen, a new laundry, a new recreation hall, a new chapel; more ground must be bought, more buildings erected; and so the process goes on. Perhaps it is not always a negligible consideration that the man who makes an institution a success is worthy of his reward; that the labour of administering a large establishment is greater, and deserves a larger salary, than that of administering a small one. We need not inquire too curiously into motives; we can see that not only is the growth of these institutions inevitable, but that they must be more and more run on the "principle of profit." The standard of accommodation is constantly rising; for more luxurious accommodation higher fees must be charged; until a stage is reached in which there is no practical difference between the management of a registered hospital and that of a licensed house.

Licensed houses form the third-class of institutions for the insane. They are avowedly managed upon the "principle of profit," and so have the advantage, at least of candour, over the registered hospitals. The difference between the two classes of institutions is that the increase of licensed houses being forbidden by law, the profit which is made goes into the hands of the proprietors; while the increase of registered hospitals being unrestricted, the profits go to extension of the institution. The interest of the managers is in each case balanced to the same extent, between keeping up the existing establishment on such a scale as to meet competition, and extracting a surplus for other purposes.

